# Conservative and functional surgery in the treatment of salivary gland tumours

**DOI:** 10.1038/s41368-019-0059-9

**Published:** 2019-08-15

**Authors:** Guangyan Yu, Xin Peng

**Affiliations:** Department of Oral & Maxillofacial Surgery, Peking University School and Hospital of Stomatology, Beijing, China

**Keywords:** Salivary gland diseases, Surgical oncology

## Abstract

The principle of modern oncological surgery is to conserve the functional organs or tissues as much as possible based on eradication of the tumour. For salivary gland tumours, conservative and functional salivary surgery, including partial sialoadenectomy as well as anatomical and functional preservation of the facial nerve, great auricular nerve, superficial musculo-aponeurotic system (SMAS), and Stensen’s duct, has become increasingly popular. In the present review, we discuss the following aspects of conservative and functional surgery in the treatment of salivary gland tumours: (i) partial superficial parotidectomy (PP) to treat benign parotid gland tumours, (ii) modification of surgical incisions to improve cosmetic results, (iii) modification of the surgical approach to decrease complications, (iv) extracardial dissection to treat benign superficial parotid tumours, (v) partial sialoadenectomy to treat benign submandibular gland tumours, and (vi) ^125^I brachytherapy to preserve facial nerves. The majority of the operated parotid or submandibular glands are preserved, and surgical complications are also decreased. Conservative and functional surgery plays a significant role in maintaining normal salivary gland function and in improving patients’ quality of life during the treatment of salivary gland tumours and thus should be further promoted.

## Introduction

Salivary gland tumours are among the most common tumours in the oral and maxillofacial regions. According to data from the seven main dental schools in China, among 69 902 cases of oral and maxillofacial tumours registered at these seven schools, 23 010 were cases of epithelial salivary gland tumours, accounting for 32.92% of all oral and maxillofacial tumours.^1^

The main function of the salivary gland is the secretion of saliva, which is critical for oral health, including protecting the oral mucosa, preventing dental carries, and maintaining the functions of mastication and speech. Surgery is the first-choice treatment for salivary gland tumours. As conventional surgical modalities, total sialoadenectomy (TS) and total superficial parotidectomy are used for benign tumours located in the submandibular gland (SMG) and the superficial lobe of the parotid gland, respectively. As a result, functional saliva secretion from these glands is lost, and varying degrees of xerostomia will occur. The parotid gland is the most common location of salivary gland tumours.^2^ Post-operative facial cosmetic results are critical for patients because of common complications that may influence the patients’ quality of life.

The principle of modern oncological surgery is to conserve the functional organs or tissues as much as possible based on eradication of the tumour. For salivary gland tumours, conservative and functional salivary surgery, including partial sialoadenectomy (PS) as well as anatomical and functional preservation of the facial nerve, great auricular nerve, superficial musculo-aponeurotic system (SMAS), and Stensen’s duct, has become increasingly popular. Partial superficial parotidectomy does not involve dissection of all branches of the facial nerve and removes less parotid tissue. Extracapsular dissection requires meticulous haemostasis and dissection of a small cuff of normal parotid parenchyma just outside the capsule of the parotid tumour, while facial nerve dissection is not performed. Gland-preserving surgery in the treatment of benign submandibular tumours is safe and preserves function without compromising local control.^3–5^ Therefore, the majority of the operated parotid or SMGs are preserved, and surgical complications are also decreased, thus improving the patients’ quality of life.

## Partial superficial parotidectomy (PP) to treat benign parotid gland tumours

Previously, conventional total superficial parotidectomy (TP) was performed to excise superficial lobe-restricted benign parotid tumours. In TP, the superficial lobe was completely resected, and the integrity of the facial nerve anatomy was maintained. The surgical goal was the complete excision of the lesion with complete anatomical and functional preservation of the facial nerve. A considerable amount of non-tumourous parotid tissue was also resected, and the intraparotid facial nerve was fully dissected to separate it from this tissue, which may disrupt Stensen’s duct, leading to a high incidence of secretory hypofunction and facial nerve paralysis or weakness.

Partial superfacial parotidectomy (PP) is defined as any procedure in which less than a superficial lobectomy is performed. During PP, only a portion of the facial nerve, i.e., the peripheral branches beyond the tumour site, is meticulously dissected, and the parotid tumour is resected with a surrounding 0.5–1.0 cm cuff of normal parotid tissue, except when the tumour abuts the plane of the facial nerve. Stensen’s duct is usually preserved in the PP unless it hinders tumour resection.^6–8^

We conducted a comparative study on salivary secretion and facial nerve function between TP and PP groups. During the study period, 268 patients with benign tumours in the superficial lobe of the parotid gland were treated, including 163 (60.8%) receiving PP and 105 (39.2%) receiving TP. After follow-up for 6 months to more than 3 years, only one patient with a Warthin’s tumour showed recurrence after PP. The TP and PP groups showed statistically similar recurrence rates (0% and 0.6%, respectively (*P* = 0.608)). The incidence of temporary facial nerve dysfunction was higher in the TP group than in the PP group (34/105, 32.4% *vs*. 29/163, 17.8%) (*P* = 0.005). Permanent palsy was more common in the TP group (4/105, 3.8%) than in the PP group (3/163, 1.8%), although the difference was not statistically significant (*P* = 0.324).^6,7^ However, statistically significant differences in the mean saliva flow rate were observed between the TP and PP groups for both the unstimulated and stimulated groups. Patients who received TP were at greater risk of parotid duct dissection and ligation than patients who received PP. With duct ligation, there is spontaneous atrophy of the glandular acini, and the parotid gland loses its secretory function. A good correlation between gland volume and saliva flow rate has been reported. Less extensive surgery, with preservation of Stensen’s duct, is important to achieve good functional restoration of the gland. Among the 165 patients in whom the duct was preserved, there were statistically significant differences in the mean saliva flow rates between the TP (*n* = 35) and PP (*n* = 130) groups. The receipt of TP was consistently associated with a lower mean saliva flow rate for both the unstimulated and stimulated groups compared with the receipt of PP.^6–8^

Frey’s syndrome is one of the post-operative complications observed after conventional parotidectomy, manifested as redness and sweating on the cheek associated with eating. In the subgroup of 83 patients who had the iodine starch test (Minor test), gustatory sweating occurred significantly (*P* < 0.001) less after PP (4/26, 15%) than after TP (52/57, 91%).^8^ Surgically, the incidence of Frey’s syndrome can be reduced by modification of the elevation of the skin flap. Conventionally, the skin flap is elevated over the level of the SMAS, which is sacrificed together with the superficial lobe of the gland. We modified the technique by elevating the skin flap under the SMAS, which is preserved together with the skin flap. The SMAS serves as the mechanical barrier between the skin and the bed of the parotid gland. A comparative study of groups receiving different surgical techniques showed that the incidence of Frey’s syndrome in patients with and without preservation of SMAS was 18% (16/89) and 41.5% (34/82), respectively. Thus, this simple and easy technique decreased the incidence of Frey’s syndrome.^7^

The great auricular nerve is located very superficially. Conventionally, the nerve is sacrificed during surgery, resulting in earlobe numbness. During functional salivary surgical techniques, the great auricular nerve in the earlobe is preserved, decreasing the incidence of earlobe numbness obviously or relieving the symptoms of numbness.^7^

The advantages of PP over TP can be summarized as follows. PP has a shorter operation time than TP because there is less surgical exposure and the procedure is easier. There is less risk of damage to the facial nerve with PP because only related branches are exposed. Facial deformity is reduced because, unlike TP, in which most of the gland is resected, which results in a depression in front of and behind the earlobe, PP involves minimal loss of the gland. The incidence of Frey’s syndrome is reduced, and importantly, most of the gland’s function is preserved.^6–8^

As indications for PP, partial superficial parotidectomy is the surgical modality of choice for primary benign tumours <2 cm in size in the superficial lobe of the parotid gland and for Warthin’s tumour in the tail of the parotid gland.^6–8^

In contrast to TP, PP is more difficult to perform and can only be performed by a very experienced surgeon who is intimately familiar with the anatomy.

Multiple primary tumours are common in patients with Warthin’s tumour, appearing as either bilateral tumours or multifocal unilateral tumours. The latter consist of either several large tumours or one large tumour with one or more “daughter tumours”. Several lymph nodes are located in the tail of the parotid gland and at the junction of the posterior border of the gland and the anterior border of the sternocleidomastoid muscle. In some cases, either microadenoma or a tumour occupying the whole node is found on microscopic examination. Thus, careful palpation during surgery should be implemented to identify the possibility of multiple tumours. The tail of the gland and the lymph nodes at the gland border should be resected to prevent recurrence.^8^

A recent study confirmed similar good results in that a low recurrence rate and a minimal risk of facial nerve weakness could be achieved with less-aggressive operations than traditional superficial parotidectomy, such as partial superficial parotidectomy. Transient facial nerve palsy is significantly less frequent after partial superficial parotidectomy (9.6%) than after total (40%) and superficial parotidectomy (28%).^9^

## Modification of surgical incisions to improve cosmetic results

Conventionally, an “S” shaped incision is used for parotidectomy, resulting in a relatively obvious facial scar. We used a face-lift incision to replace the “S”-shaped incision in selected patients. When a benign tumour was located in the upper part of the superficial lobe of the parotid gland, a pre-auricular incision within the hair-line was used, while a retro-auricular incision within the hair-line was used when the tumour was located in the tail of the parotid gland. All the selected patients were treated successfully. The wound healed uneventfully. Compared with the patients receiving the conventional “S”-shaped incision, in patients who received the face-lift incision, there was no difference in the evaluation index of operating time, the amount of bleeding, or the rate of post-operative complications (*P* > 0.05), while patients’ satisfaction among those who received the modified incision was significantly higher (*P* *<* 0.01). The incision was hidden and provided satisfactory cosmetic results in patients undergoing conservative parotidectomy.^7,10,11^ A recent study showed that a modified V-shaped incision for parotidectomy was safe and feasible for small-to-medium benign parotid tumours. The approach could be a possible option for patients with high cosmetic demand.^12^

## Modification of the surgical approach to decrease complications

The surgical approach used to resect a large tumour in the deep lobe of the parotid gland usually involves osteotomy at the mandibular angle. The inferior alveolar nerve is cut, resulting in lower lip numbness. The tumour cannot be exposed well, which may result in breaking the tumour, resulting in recurrence because of tumour cell seeding. Post-operative radiotherapy may be necessary if the tumour is malignant. In this situation, radiation-induced osteonecrosis may occur because the location of the osteotomy is within the radiotherapy field. We changed the location of the osteotomy to the premental foramen area. Fifty-one patients subjected to mandibulotomy of the premental foramen area were reviewed and followed up for 3 months to six years (mean = 26 months). Tumours of the deep lobe of the parotid gland could be exposed clearly and resected radically. Compared with conventional osteotomy at the mandibular angle, with premental foramen mandibulotomy, the surgical complications were reduced. The new surgical approach could expose the tumour better, preserve the integrity of the inferior alveolar nerve to avoid lower lip numbness, and prevent radiation-induced osteonecrosis because the osteotomy location was beyond the radiotherapy field.^7,13^

## Extracapsular dissection to treat benign superficial parotid tumours

We investigated the clinicopathological characteristics and management of parotid pleomorphic adenomas closely abutting the facial nerve. In 20 cases (21 sides) of parotid gland pleomorphic adenoma, the facial nerve closely abutted the tumour during surgery. The nerve was carefully dissected and preserved, and the tumour was removed with the parotid gland. The capsular structure of the tumour in the location where the facial nerve was closely abutted was examined histopathologically. The patients were followed up after treatment. The results showed that a complete capsule was observed in the location of the tumour closely abutting the facial nerve. A very thin but complete capsule was shown in five cases, and infiltrated tumour nodules within the capsule were present in three cases. After a follow-up of >3 years in 76.2% of the patients, no tumour recurrence occurred in the patients without tumour spillage during surgery, while tumour recurrence occurred in one patient with tumour spillage. These results indicated that if there was a thin but complete capsule where the facial nerve closely abutted the parotid pleomorphic adenoma, careful dissection and preservation of the facial nerve were reliable. Avoiding tumour spillage during surgery is vital to prevent recurrence via tumour seeding. These results provide a histopathological basis for the extracapsular dissection of benign parotid tumours.^14^

Extracapsular dissection could also be used in patients with clinically benign lumps with superficial, clear boundaries and movable characteristics.^15^ From 1952 to 1992, 630 patients were treated in the Center of Salivary Gland Diseases Christie Hospital, including 491 patients who received extracapsular dissection and 139 patients who received conventional superficial parotidectomy. After a long follow-up (mean = 15 years), the recurrence rates were statistically similar at 1.6% and 1.8% in the extracapsular dissection and conventional superficial parotidectomy groups, respectively, while surgical morbidity was obviously reduced.^15,16^ We also treated 49 patients with benign superficial parotid tumours using extracapsular dissection; no tumour recurrence was found after at least 1 year of follow-up. With accumulating surgical experience, this technique of conservative and functional surgery has become more acceptable worldwide. However, the selection of indications and the performance of experienced surgeons are critical to prevent tumour recurrence.

## Partial sialoadenectomy (PS) to treat benign submandibular gland (SMG) tumours

The SMG is responsible for 60%–65% of resting salivary flow.^17^ The conventional treatment for benign tumours in the SMG is extirpation of the entire gland along with the tumour,^18,19^ resulting in decreased resting saliva production.^18,20^ Retaining the vascular and ductal system integrity of the remaining SMG is crucial for a successful outcome. Previously, we studied the microanatomy of the human SMG by perfusing methacrylate to form resin casts of the blood vessels and ducts, which showed a tree-like shape with similar structures of blood vessels and ducts at each level in the lobules.^21^ This tree-like structure provides a solid anatomical basis for partial submandibular sialoadenectomy. Moreover, Roh and Park^5^ and Min et al.^22^ reported a series of 20 patients with pleomorphic adenoma in the SMG treated using gland-preserving surgery. The patients had symmetrical facial contours without defects in the operated submandibular triangle. Gland-preserving surgery in patients with benign SMG tumours can preserve salivation and reduce surgical morbidity and operating time, with good cosmesis but without compromising local control.

We performed a prospective study to evaluate the effectiveness and safety of PS for benign tumours compared with conventional TS in 31 consecutive patients with a preoperative diagnosis of benign tumour in the SMG.^18^ Eleven patients were treated with PS and 20 with TS. Salivary gland function and surgery-related complications were assessed. No difference in resting saliva flow was found between the two groups before surgery; however, the resting saliva flow was significantly higher in the PS group than in the TS group 1 year after surgery (*P* = 0.009). With regard to complications, there was comparatively less facial deformity in the PS group than in the TS group. None of the 31 patients experienced recurrence during follow-up (range: 41–82 months).^18^ This indicated that modification to SMG surgery is consistent with the principle of functional and minimally invasive salivary gland surgery. This technique represents a good choice to manage benign tumours of the SMG in certain cases.

It is important to master the indications and contraindications of PS. For example, malignancy of the SMG is a contraindication to the use of this technique. In our experience, the benign tumour’s location is critical when selecting the modality of PS. If the tumour is located in the centre of the SMG, the vascular and ductal system integrity of the remaining SMG will be damaged after PS. Wharton’s duct will be damaged if the tumour is located close to the duct. A benign tumour located in the lateral part of the SMG commonly projects into the normal surrounding tissues; in this case, it is not difficult to perform a PS. Therefore, the tumour size is not a limitation for this technique. We suggest the following indications for PS: (1) primary benign tumour in the SMG and (2) tumour located in the lateral part of the SMG and far from Wharton’s duct. The suggested contraindications of this technique are (1) malignancy of the SMG and (2) a tumour located in the central part of the SMG or close to Wharton’s duct. Based on the importance of tumour location, a preoperative computed tomography scan is essential to locate the tumour in the SMG.^18^

## ^125^I brachytherapy to preserve facial nerves

Routinely, malignant parotid gland tumours are extensively excised together with the parotid gland. The facial nerve is always sacrificed if it is adherent to the tumour. Facial nerve resection results in drooping of the angle of the mouth and difficulty in closing the eye and raising the eyebrow, which seriously affects the patient’s quality of life. Facial nerve preservation may compromise radical surgery if the tumour has invaded the facial nerve. In this situation, post-operative radiotherapy with 40–60 Gy is routinely advised. However, external radiotherapy of the head and neck region is associated with severe complications, such as extensive oral mucosal ulceration and osteoradionecrosis of the jaw. ^125^I seed brachytherapy is an alternative method to external radiotherapy. Surgery for malignant parotid gland tumours combined with ^125^I seed implant brachytherapy and preservation of the facial nerve was conducted by Zhang’s et al.^23^ ^125^I seeds were implanted into the target area intra- or post-operatively. None of the 12 patients had tumour recurrence during the follow-up period of 50–74 months (median = 66 months). Normal facial nerve function recovered by 6 months post-operatively in all patients. Therefore, a limited surgical resection combined with ^125^I seed implant brachytherapy represents an alternative treatment for the local control of malignant parotid gland tumours with facial nerve preservation.^23^

In summary, conservative and functional surgery plays a significant role in maintaining normal salivary gland function and in improving patients’ quality of life during treatment of salivary gland tumours and thus should be further promoted. However, mastering the indications for these techniques and performance by experienced surgeons are critical.
